# The Impact of Lifetime Alcohol and Cigarette Smoking Loads on Multiple Sclerosis Severity

**DOI:** 10.3389/fneur.2019.00866

**Published:** 2019-08-13

**Authors:** Andrei Ivashynka, Massimiliano Copetti, Paola Naldi, Sandra D'Alfonso, Maurizio A. Leone

**Affiliations:** ^1^Department of Health Sciences, University of Eastern Piedmont, Novara, Italy; ^2^Neurology Unit, Department of Medical Sciences, Fondazione IRCCS Casa Sollievo della Sofferenza, San Giovanni Rotondo, Italy; ^3^Unit of Biostatistics, Fondazione IRCCS Casa Sollievo della Sofferenza, San Giovanni Rotondo, Italy; ^4^Department of Neurology, University Hospital “Maggiore della Carità”, Novara, Italy; ^5^Interdisciplinary Research Center of Autoimmune Diseases, University of Eastern Piedmont, Novara, Italy

**Keywords:** multiple sclerosis, multiple sclerosis severity score, lifetime alcohol load, lifetime cigarette smoking load, risk factors, cross-sectional study

## Abstract

**Background:** The association between lifestyle factors and Multiple Sclerosis (MS) disease severity and progression has been investigated to a lesser extent compared with susceptibility to the disease.

**Objective:** We aimed to assess the impact of lifetime alcohol and cigarette smoking load on MS severity.

**Methods:** Design: a cross-sectional study. Three hundred fifty-one patients consecutively admitted to the Department of Neurology were asked to complete the “Questionnaire of Lifestyle” (part of the European Prospective Investigation into Cancer and Nutrition project). An estimation of the cumulative lifetime cigarette smoking and alcohol load was calculated as the weighted sum of the mean number of cigarettes smoked and standard alcoholic drinks consumed per day at different ages. The measure of exposure was expressed in terms of pack-year and drink-year. Disease severity was estimated by the Multiple Sclerosis Severity Score (MSSS). Logistic regression analyses were performed using MSSS (first tertile vs. third tertile) as the outcome.

**Results:** The median MSSS was higher (3.2 vs. 2.3, *p* = 0.002) in ever- vs. never-smokers, but we did not find a difference between ever- and never-drinkers (2.7 vs. 2.8, *p* = ns). Ever-smokers were almost twice as likely to fall in the upper MSSS tertile than never-smokers. Ever-drinkers did not show a statistically significant association between alcohol intake and MS severity. The risk of falling in the worst MSSS tertile for smokers was 10.81 (2.0–58.48; *p* < 0.01) if they were never-drinkers, whereas it was only 1.65 (0.89–3.03, *p* = 0.11) if they were also drinkers. On the other side, the risk of falling in the worst MSSS tertile for drinkers did not change as much, whether they also were smokers (0.46; 0.13–1.65; *p* = 0.23) or not (1.49; 0.55–4.04, *p* = 0.43).

**Conclusions:** Cigarette smoking, unlike alcohol consumption, is associated with MS severity. Alcohol consumption may attenuate the effect of smoking on disease severity, acting as an effect modifier. The biological background of this effect is unknown. The limitations of our study are mostly due to its cross-sectional design.

## Introduction

Multiple sclerosis (MS) is an autoimmune disease of the central nervous system with a heterogeneous clinical course, characterized by multifocal inflammatory demyelination and secondary axonal degeneration, in which the interaction between environmental and genetic protective and risk factors leads to the chronic activation of immune cells, and neuronal injury (1, 2).

There is ample evidence that lifestyle habits, such as alcohol use and cigarette smoking, are risk factors for the development of autoimmune diseases. Cigarette smoking has been associated with susceptibility to MS (3) as well as to other autoimmune diseases (4), whereas the effect of alcohol is still controversial (5). The association of lifestyle factors with MS disease severity and progression is less explored compared with susceptibility to the disease.

Potentially modifiable lifestyle factors, such as alcohol consumption and cigarette smoking might play a role in MS progression, and this is essential from a clinical as well as an etiological point of view. In some studies the harmful effect of cigarette smoking on the course of already established MS was proved (6–9), whereas only a handle of studies had evaluated the relationship between alcohol intake and disease severity so far (5, 9, 10), and none of them had studied the combined effect of these two factors.

Since both alcohol use and cigarette smoking are common chronic risk factors, their long-term effects may depend on cumulative lifetime exposure, even in low to moderate doses. That is why the study goal was to evaluate the association between lifetime alcohol consumption and lifetime cigarette smoking with MS severity.

## Methods

### Patients and Data Collection

The research was designed as a cross-sectional study. It was approved by the Ethics Committee of the University Hospital “Maggiore della Carità” in Novara (Italy). Patients followed at the MS Center of the Department of Neurology were consecutively recruited between 2011 and 2012. It is a first-referral MS Center, covering a catchment area of around 400,000 populations. MS patients are referred by other neurologists and General Practitioners and are regularly followed with twice yearly visits. Of the 356 patients who were asked to participate, only five refused to take part in the study. Written informed consent was obtained from 351 participants. All cases were diagnosed by neurologists according to the McDonald criteria (11). Patients were enrolled during follow-up visits scheduled out of a relapse. Demographics (date of birth, age, sex, profession, marital status, education) and clinical data (disease course, date of onset and diagnosis, EDSS) were collected. Cigarette smoking and alcohol consumption histories were evaluated with the “Questionnaire of Lifestyle” which is part of the European Prospective Investigation into Cancer and Nutrition project (EPIC) study (12). All the patients were asked to fill the questionnaire during the clinic visit. In case of difficulty, they were free to ask for help one interviewer who was blinded to the clinical history and severity evaluation.

### Disease Severity Examination

Disease severity was estimated through the Expanded Disability Status Scale (EDSS) and the Multiple Sclerosis Severity Score (MSSS). The MSSS corrects EDSS for disease duration, allowing to compare an individual's disability with the distribution of scores in cases having similar EDSS scores. The MSSS score (range 0–9.9) was calculated, according to Roxburgh et al. (13).

### Exposure Assessment

Cigarette smoking and alcohol consumption data were collected at recruitment. All subjects were asked to report their cigarette smoking status at the time of the interview (never, former, or current smoker). *Never-smokers* were patients who had smoked <100 cigarettes up to the time of the interview (14). *Former smokers* were patients who had smoked >100 cigarettes and had stopped smoking at least 6 months before the time of the interview. Former smokers were asked to state the age when they started and then quit smoking. *Current smokers* were those who had smoked >100 cigarettes and were still smoking at the time of the interview. *Ever*-*smokers* (former or current) were asked to quantify the number of cigarettes per day smoked at recruitment and four age periods (20, 30, 40, and 50 years). For each age period, we calculated the mean number of cigarettes smoked per day based on the questionnaire information and the number of years spent smoking. The cigarette smoking duration (years) was calculated as the difference between age at recruitment (for current smokers) or age at cigarette smoking cessation (for former smokers), and age at start smoking.

An estimation of the *cumulative lifetime smoking load* was calculated as the weighted sum of the mean number of cigarettes that were smoked per day at different ages, including at the time of recruitment, with weights equal to the cigarette smoking duration for each age period. The measure of exposure was expressed in *pack-year*, defining a pack as containing 20 cigarettes.

Similarly, detailed information was obtained regarding alcohol consumption during different age periods up to the participants' current age. The alcohol drinking status at the time of the interview (never, former, or current drinker) was assessed. We defined as *never-drinkers* those who had drunk less than one standard alcoholic drink/month. *Former drinkers* were patients who had drunk one or more standard alcoholic drinks/month and had stopped drinking at least 6 months before the interview. *Ever-drinkers* (former or current) were asked to state the number of alcoholic drinks per day by type of beverage (wine, beer and spirits), at the time of interview and four age periods (20, 30, 40, and 50 years). The method used to calculate the cigarette smoking duration was also used to calculate the number of years of alcohol consumption. An Italian standard alcoholic drink (standard alcoholic unit) contains approximately 12 grams of pure ethanol (15), corresponding to a small glass of wine (125 ml), a can of beer (330 ml), or a shot of spirits (40 ml).

In analogy with the *cumulative lifetime smoking load*, a *cumulative lifetime alcohol drinking load* (*drink-year*) was calculated as the weighted sum of the mean number of standard alcoholic units per day at different age periods with weights equal to the number of years spent drinking for each age period for each type of beverage, and in total (aggregating all kinds of drinks).

### Statistical Analysis

Patients' baseline characteristics are reported as median with range and frequencies for continuous and categorical variables, respectively. According to their smoking and drinking status at the time of the interview, subjects were classified into *never, former* or *current smokers*/*drinkers*. *Ever*-(*former* and *current*) smokers/ever-drinkers were categorized into two groups (*lower load* vs. *higher load*) according to the median value of the *pack-year* or *drink-year* distribution.

A new categorical variable was defined to explore the combination of smoking and drinking habits. The four categories were: (1) never-smokers/never-drinkers; (2) never-smokers/ever-drinkers; (3) ever-smokers/ever-drinkers; (4) ever-smokers/never-drinkers.

MSSS was converted into a trichotomous categorical variable, based on tertiles of the distribution, and logistic regression analyses were performed using MSSS <1.8 (first tertile) vs. >3.9 (third tertile) as the outcome, thus comparing the two extremes of the MSSS distribution.

Normal distribution assumption was checked using the Shapiro-Wilks test and the Q-Q plot. Comparisons between two or more groups were tested using the Chi-square test for categorical variables and Student's *T*-test or ANOVA analysis for continuous variables. Non-parametric Mann-Whitney and Kruskal-Wallis tests were used when appropriate.

The association between smoking and drinking habits and disease severity was evaluated using a univariable and multivariable logistic regression model that was adjusted for age, sex, and education. Risks were reported as odds ratios (OR) along with their 95% confidence intervals (CI). A sensitivity analysis was conducted considering the three types of beverages (wine, beer, and spirits) separately.

To assess the possibility of an effect modification, we evaluated the risk for ever-smokers of being in the upper (worst) MSSS tertile in the two subsets of ever-drinkers and never-drinkers, separately; on the other side, we assessed the risk of ever-drinkers of being in the worst MSSS tertile in the two subgroups of ever-smokers and never-smokers.

A *p*-value <0.05 was considered as statistically significant. Analyses were performed using SAS®, version 9.3 (SAS Institute, Cary, NC).

## Results

We recruited 351 patients, 235 women and 116 men, with a sex ratio of 2:1. The mean age was 33.0 ± 10.1 years at MS onset, and 44.1 ± 11.7 years at recruitment. The mean disease duration at recruitment was 11.1 ± 8.1 years. The other demographic and clinical characteristics are presented in Table 1. Only three patients began to smoke after being diagnosed with MS, and 26 patients began to consume alcohol after the MS diagnosis.

**Table 1:** Demographical, clinical and exposure characteristics of the MS sample.

**Variable**	**Category**	**Median (min-max) or frequency (%)**
*N*		351
Age at MS onset (years)		32 (6.82–64.35)
Education (years)		13.00 (5.00–24.00)
Disease duration (years)		10.00 (1.00–48.00)
EDSS		2.00 (0.00–8.00)
MSSS		2.70 (0.05–9.86)
The clinical form of MS	Relapsing-remitting	266 (75.78)
	Progressive (primary and secondary)	85 (24.22)
Cigarette smoking		
status	Current Smokers	92 (26.21)
	Never-smokers	161 (45.87)
	Former smokers	98 (27.92)
Lifetime Cigarette Smoking Load (pack-year)^*^		10.63 (0.10–78.37)
Alcohol drinking status(general)	Current drinker	256 (72.93)
	Never-drinker	68 (19.37)
	Former drinker	27 (7.69)
Wine	Current drinker	218 (62.11)
	Never-drinker	108 (30.77)
	Former drinker	25 (7.12)
Beer	Current drinker	181 (51.57)
	Never-drinker	134 (38.18)
	Former drinker	36 (10.26)
Spirits	Current drinker	125 (35.61)
	Never-drinker	165 (47.01)
	Former drinker	61 (17.38)
Lifetime Alcohol Load(drink-year)^**^		7.49 (0.36–394.09)

* *Ever-(current and former) smokers (n = 190)*.

** *Ever-(current and former) drinkers (n = 283)*.

Table 2 shows unadjusted comparisons between never and ever-smokers. Ever-smokers were older, more frequently males and had a lower education level than never-smokers. As expected, ever-smokers tend to drink more than people who had never smoked. Finally, the univariable analyses showed that the disease, in term of MSSS, was more severe in people who had smoked than in never-smokers. The association between MSSS and smoking remained significant in the subsample of drinkers (data not shown, *p* = 0.037). No significant differences in disease severity were found between smokers with lower and higher smoke loads.

**Table 2:** Subgroup comparisons according to cigarette smoking status.

**Variable**	**Category**	**Never-smokers**	**Ever-smokers**	***p*-value**	Lower smoke load^*^	Higher smoke load^*^	***p*-value**
***N***		**161**	**190**		**95**	**95**	
		**Median (min-max) or frequency (%)**			**Median (min-max) or frequency (%)**		
Age at MS onset (years)		30.11 (6.82–64.35)	34.19 (12.21–60.70)	0.005	31.53 (12.21–60.70)	35.95 (12.76–60.70)	0.013
Education (years)		13.00 (5.00–24.00)	11.00 (5.00–23.00)	0.001	12.00 (5.00–23.00)	11.00 (5.00–19.00)	0.044
Sex	Female	120 (74.53)	115 (60.53)	0.005	67 (70.53)	48 (50.53)	0.005
	Male	41 (25.47)	75 (39.47)		28 (29.47)	47 (49.47)	
Disease duration (years)		10.00 (1.00–48.00)	9.00 (1.00–41.00)	0.586	8.00 (1.00–33.00)	11.00 (1.00–41.00)	0.001
MSSS		2.33 (0.05–9.86)	3.21 (0.13–9.68)	0.002	3.44 (0.16–9.59)	3.10 (0.13–9.68)	0.982
Clinical form of MS	Relapsing-remitting	128 (79.50)	138 (72.63)	0.127	76 (80.00)	62 (65.26)	0.020
	Progressive forms	33 (20.50)	52 (27.37)		19 (20.00)	33 (34.74)	
Alcohol drinking status	Current drinker	109 (67.70)	147 (77.37)	0.076	77 (81.05)	70 (73.68)	0.332
	Never-drinker	35 (21.74)	33 (17.37)		15 (15.79)	18 (18.95)	
	Former drinker	17 (10.56)	10 (5.26)		3 (3.16)	7 (7.37)	
Lifetime alcohol Load (drink-year)		2.65 (0.00–101.95)	8.00 (0.00–394.09)	<0.001	6.85 (0.00–86.05)	11.47 (0.00–394.09)	0.173

* *The two groups (lower vs. higher Lifetime Cigarette Smoking Load) were categorized according to their median value*.

Table 3 shows unadjusted comparisons between never and ever-drinkers. As expected, alcohol consumption was more frequent in males than in females. No difference in disease severity based on MSSS scores was noted when comparing ever with never-drinkers. MSSS was significantly lower for patients with lower alcohol load than for those with higher alcohol load. The association between MSSS and alcohol load remained significant in the sub-sample of smokers (data not shown, *p* = 0.026). However, the other predictive factors (age, sex, age at onset, smoking) were unequally distributed in the two groups.

**Table 3:** Subgroup comparisons, according to the alcohol drinking status.

**Variable**	**Category**	**Never-drinkers**	**Ever-drinkers**	***p*-value**	Lower alcohol load^*^	Higher alcohol load^*^	***p*-value**
***N***		**68**	**283**		**141**	**142**	
		**Median (min-max) or frequency (%)**			**Median (min-max) or frequency (%)**		
Age at MS onset (years)		32.75 (12.76–64.35)	32.03 (6.82–60.70)	0.653	29.88 (6.82–56.28)	35.13 (14.83–60.70)	<0.001
Education (years)		13.00 (5.00–19.00)	13.00 (5.00–24.00)	0.659	13.00 (5.00–24.00)	11.00 (5.00–22.00)	0.002
Sex	Female	59 (86.76)	176 (62.19)	<0.001	103 (73.05)	73 (51.41)	<0.001
	Male	9 (13.24)	107 (37.81)		38 (26.95)	69 (48.59)	
Disease duration (years)		12.50 (1.00–34.00)	9.00 (1.00–48.00)	0.042	8.00 (1.00–45.00)	10.00 (1.00–48.00)	0.001
MSSS		2.83 (0.26–9.20)	2.65 (0.05–9.86)	0.465	2.33 (0.05–9.86)	3.18 (0.05–9.68)	0.014
Form of MS	Relapsing-remitting	48 (70.59)	218 (77.03)	0.272	119 (84.39)	99 (69.72)	0.004
	Progressive forms	20 (29.41)	65 (22.97)		22 (15.61)	43 (30.28)	
Cigarette smoking status	Current smokers	19 (27.94)	73 (25.80)	0.317	29 (20.57)	44 (30.99)	<0.001
	Never-smokers	35 (51.47)	126 (44.52)		84 (59.57)	42 (29.58)	
	Former smokers	14 (20.59)	84 (29.68)		28 (19.86)	56 (39.44)	
Lifetime cigarette smoking load (pack-year)		0.00 (0.00–32.80)	1.70 (0.00–78.37)	0.483	0.00 (0.00–35.70)	6.58 (0.00–78.37)	<0.001

* *The two groups (lower vs. higher Lifetime Alcohol Load) were categorized according to their median value*.

**Figure 1:**
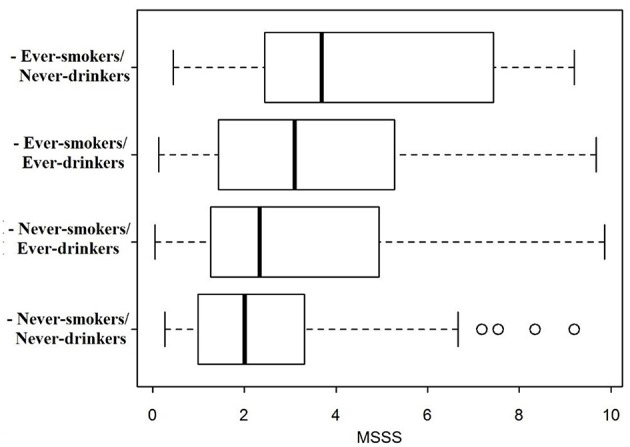
MSSS distribution boxplot in subgroups according to smoking and drinking habits.

The MSSS distribution was compared across the different categories according to drinking/smoking habits (Figure 1). MS severity was lowest in patients who never had drunk nor smoked, highest in patients who had smoked but had never drunk, and intermediate for patients who had only drunk or had both drunk and smoked.

Logistic regression models after controlling for age, sex, and education were estimated to confirm these results, comparing the extremes of the MSSS distribution (upper vs. lower tertile) (Table 4). Ever-smokers were almost twice as likely to fall in the upper than in the lower MSSS tertile compared with never-smokers, whereas no difference was found for drinking habits (model A and B). In both models of ever-smokers and ever-drinkers (model C and D) no significant association was found between categories of lifetime load and disease severity. However, patients who had only smoked were six times more likely to fall in the upper rather than the lower MSSS tertile compared with patients who never had drunk or smoked, but alcohol consumption seems to mitigate this association (model E).

**Table 4:** Age, sex, and education-adjusted logistic regression models: upper MSSS tertile vs. lower MSSS tertile (*N* = 230).

			**OR**	**95% CI**	***p*-value**
Model A	Cigarette smoking status	Ever-smokers (*n* = 120) vs. Never-smokers (*n* = 110)	1.89	1.08–3.30	0.026
Model B	Alcohol drinking status	Ever-drinkers (*n* = 190) vs. Never-drinkers (*n* = 40)	0.98	0.47–2.03	0.961
Model C	Lifetime cigarette smoking load (pack-year)^*^	Lower LCSL (*n* = 61)	2.64	1.33–5.24	0.005
		Higher LCSL (*n* = 59)	1.30	0.65–2.61	0.465
		Never-smokers (*n* = 110)	1.00		
Model D	Lifetime alcohol load (drink-year)^**^	Lower LAL (*n* = 87)	0.86	0.38–1.92	0.711
		Higher LAL (*n* = 103)	1.10	0.50–2.42	0.808
		Never-drinkers (*n* = 40)	1.00		
Model E	Four categories	Never-smokers/ever-drinkers (*n* = 87)	1.75	0.64–4.81	0.278
		Ever-smokers/Ever-drinkers (*n* = 103)	2.54	0.94–6.88	0.067
		Ever-smokers/Never-drinkers (*n* = 17)	6.62	1.54–28.33	0.011
		Never-smokers/Never-drinkers (*n* = 23)	1.00		

* *Lower vs. higher Lifetime Cigarette Smoking Load (Ever-smokers): OR = 2.04; 95% CI = 0.91–4.57*.

** *Lower vs. higher Lifetime Alcohol Load (Ever-drinkers): OR = 0.78; 95% CI = 0.41–1.49*.

*LAL, Lifetime Alcohol Load; LCSL, Lifetime Cigarette Smoking Load*.

To further explore the combined effect of the two exposures, we stratified patients by drinking and smoking habits, adjusting for sex and age. The risk of falling in the worst MSSS tertile for smokers was 10.81 (2.0–58.48; *p* < 0.01) if they were never-drinkers, whereas it was only 1.65 (0.89–3.03, *p* = 0.11) if they were also drinkers. On the other side, the risk of falling in the worst MSSS tertile for drinkers did not change as much, whether they also were smokers (0.46; 0.13–1.65; *p* = 0.23) or not (1.49; 0.55–4.04, *p* = 0.43).

## Discussion

The results of this study confirm those of several observational studies suggesting that cigarette smoking may exert an influence on the MS course. Cohort studies of Clinically Isolated Syndrome patients concluded that conversion to clinically definite MS and disease progression were more rapid in ever-smokers compared to never-smokers (8, 16). Hernan et al. found that cigarette smoking increased the risk of conversion from relapsing-remitting to secondary progressive MS (17). A Swedish study of self-reported data from MS patients found that, after a median of 6 years, ever-smokers were more likely to have progressive disease compared to never-smokers. Moreover, the effects on the rate of conversion to progressive MS and earlier age of progression onset were highest in those who began smoking before the age of 15. Early onset of regular smoking also associated with a higher percentage of primary progressive MS when compared with never-smokers (18).

Our study found no significant difference in MS severity between ever-drinkers and never-drinkers. MSSS values were lower among people who had drunk less alcohol during their lifetime compared to those who had consumed higher amounts of alcohol. To date, only a few studies have evaluated a possible effect of alcohol consumption on disease severity in MS. Alcohol consumption was associated with less total disability in cross-sectional studies that used different outcomes and MS severity end-points: EDSS (19, 20), MSSS (20), Patient-Determined Disease Steps (21), Incapacity Status Scale Scores (22), and reaching EDSS 6 (23, 24). Two studies showed an inverse dose-response association between alcohol consumption and disability measured with EDSS (19, 21), while two other cross-sectional studies did not find any association (25, 26). In addition to the inherent limitations of the cross-sectional study design, these studies were affected by selection bias, as were enrolled persons from MS patient associations or online, or used convenience samples (19, 22, 23). Some studies had a low response rate (19, 25), and did not take into account possible confounders (age, sex, disease duration, type of therapy, Body Mass Index, other chronic diseases). The only prospective study (9) found that ex-consumers of alcohol had a lower risk of MS progression than current consumers, but after stratifying by sex, no significant differences were found among non-consumers, ex-consumers and current alcohol consumers. In previous studies, alcohol consumption was ascertained in terms of quantity/frequency (19, 21, 23, 26), duration of consumption (20), or using validated questionnaires (9, 22, 25, 26). No experimental clinical study has been conducted because of ethical limitations.

The most remarkable result of our study is that ever-smokers/ever-drinkers MS patients were only twice as likely to be in the upper MSSS tertile compared to never-smokers/never-drinkers, whereas ever-smokers who never drank had a 6-fold chance of being in the same upper (worst) tertile. The median MSSS was also higher in ever-smokers/never-drinkers than in ever-smokers/ever-drinkers. The interplay of the two exposures is intricate: a confounding effect was improbable since, after stratification for drinking habits (not/yes), the OR for ever-smokers was higher in the never-drinker stratum compared to the ever-drinker stratus (10.8 vs. 1.7). It points to the possibility that alcohol acts as an effect modifier of smoking exposure. Insufficient sample size could explain the partial overlap of the CIs.

A similar observation (alcohol-induced attenuation of the effect of smoking) comes from two large studies on MS *susceptibility*, in which both incident and prevalent MS cases were asked to report their alcohol habits prior to or close to the time of disease onset compared to age- and sex-matched controls (2). Thus, a possible attenuation of the effect of smoking by alcohol use could occur either before the disease onset or during the disease, as shown for the first time in our study.

The biological background of this effect is unknown, and we can only speculate upon it. An interplay between alcohol use and cigarette smoking could occur at different levels. Firstly, both alcohol and smoke are potential modulators of the gastric and intestinal microbiome (27), which may play a critical role in the development or progression of the disease by modulating the inflammatory process (28). Secondly, since both alcohol and smoke might be able to modulate several components of the immune system, the interaction could happen at this level leading to altered inflammatory responses. Many immunological and inflammatory effects of alcohol are dose-dependent (29). Thirdly, alcohol and its metabolites directly affect the central nervous system. The effect of alcoholic beverages depends on many factors, including the properties of the drinks, the presence of additional components, drinking frequency and patterns, dose, and setting of use. Thus, the balance between harmful effects due to the ethanol itself and possible protective effects due to other substances such as resveratrol or flavonoids varies widely. Therefore, future studies on alcohol use and MS risk or severity are needed to explore also the possible dose-effect of alcohol exposure and should take into account the type of beverage and the total alcohol load. We performed an analysis (data not shown) by kind of alcoholic drink, but the study sample size was not sufficiently powered for such subgroup analysis.

The limitations of this study are mostly due to its cross-sectional design, where the outcomes of interest and exposures are carried out at the same point in time and do not indicate the sequence of events, whether exposure determines the severity or vice-versa. For this reason, it is not possible to infer causality. Those with a more severe disease progression might have modified their alcohol and smoke habits, and this might have influenced their report of previous exposures. Furthermore, we cannot exclude a recall bias using lifestyle questionnaires for data collection. Our study did not have enough power for subgroup analyses by sex and types of MS, and we could not evaluate the influence of unmeasured confounders, such as BMI, or vitamin D.

On the other hand, our study does present some strengths. Selection bias was minimized because patients were enrolled at a first-referral Center serving most patients of its catchment area, recruitment was consecutive, and the duration of the recruitment period allowed us to recruit almost all the patients followed-up at the MS Center. Recall bias is unavoidable with this type of study, but patients were unaware of the study hypothesis, questionnaires were self-administered, and the helping interviewer was blinded to neurological status. Using the lifetime alcohol load (*drink-year*) and cigarette smoking load (*pack-year)* to measure exposure, we were able to study the lifelong cumulative effect of both exposures and not only the amount of exposure at the time of the interview or immediately before. Also, it was examined the impact of smoke and alcohol on the course of MS simultaneously.

In conclusion, this study adds to the knowledge of the association of two common harmful lifestyle factors with MS severity, supporting the need for further investigation into their role in disease progression and their possible interaction with other lifestyle factors. Given the many potential confounders and the possibility that different factors act in different stages of the disease, further studies need to be large enough to consider all the potential confounders and differentiate between risk factors for susceptibility to the disease and those predictive for severity and MS progression (1). Cumulative measures, such as *pack-year* and *drink-year* should be used for estimating the lifetime effect of alcohol use and cigarette smoking on the course of MS. This study results also have practical relevance, actively supporting abstinence from smoking in MS patients, whereas to date, no clear recommendation may be issued for social drinking to patients with MS. Since patients with MS exhibit numerous adverse health behaviors (30), attention to the management of potentially modifiable lifestyle factors for slowing progression in patients with MS is worthwhile.

## Data Availability

The raw data supporting the conclusions of this manuscript will be made available by the authors, without undue reservation, to any qualified researcher.

## Ethics Statement

The work was approved by the Ethics Committee of the University Hospital Maggiore della Carità, Novara, Italy.This neuroepidemiological research was based on the observational (cross-sectional) study design.

## Author Contributions

AI: study design, recruitment, and examination of patients, data collecting, management, and analysis, final approval of the version to be published, drafting the paper, agreement to be accountable for all aspects of the work in ensuring that questions related to the accuracy or integrity of any part of the work are appropriately investigated and resolved. MC: study design, data analysis, data interpretation, and writing. PN: acquisition of data for the work (in enrolling the sample of multiple sclerosis patients followed at the Department of Neurology of the Maggiore della Carita hospital, to collect patient-related information and informed consents, to perform disease severity examination. Revised the paper critically for important intellectual content (results of analysis and discussion). Dubble-check the paper organization and the accuracy of the provided information, agree with conclusions. Final approval of the version to be published and decide to be accountable for all aspects of the work in ensuring that questions related to the accuracy of the integrity of any part of the work are appropriately investigated and resolved. SD'A: contribution to the conception of the study, final approval of the version of the manuscript to be published, and revising critically the research and the manuscript. ML: study design, recruitment, data management, and analysis, drafting the paper, final approval of the version to be published, agreement to be accountable for all aspects of the work in ensuring that questions related to the accuracy or integrity of any part of the work are appropriately investigated and resolved.

### Conflict of Interest Statement

The authors declare that the research was conducted in the absence of any commercial or financial relationships that could be construed as a potential conflict of interest. The reviewer KM declared a past co-authorship with one of the authors ML to the handling editor.
